# The Utility of Intraventricular Pressure Gradient for Early Detection of Chemotherapy-Induced Subclinical Cardiac Dysfunction in Dogs

**DOI:** 10.3390/ani11041122

**Published:** 2021-04-14

**Authors:** Katsuhiro Matsuura, Kenjirou Shiraishi, Ahmed S. Mandour, Kotomi Sato, Kazumi Shimada, Seijirow Goya, Tomohiko Yoshida, Pitipat Kitpipatkun, Lina Hamabe, Akiko Uemura, Zeki Yilmaz, Mayumi Ifuku, Takeshi Iso, Ken Takahashi, Ryou Tanaka

**Affiliations:** 1 VCA Japan Shiraishi Animal Hospital, Saitama 350-1304, Japan; k.matsuura.vet@gmail.com; 2 Veterinary Surgery, Tokyo University of Agriculture and Technology, Tokyo 183-0054, Japan; kenkenvet@gmail.com (K.S.); sleeping_3straycat@yahoo.co.jp (K.S.); ruiyue1221@gmail.com (K.S.); seijirow.goya@gmail.com (S.G.); tomohiko7731-yoshida@yahoo.co.jp (T.Y.); pitipat_ki@rmutto.ac.th (P.K.); linahamabe@googlemail.com (L.H.); 3 Department of Animal Medicine (Internal Medicine), Faculty of Veterinary Medicine, Suez Canal University, Ismailia 41522, Egypt; 4 Department of Veterinary Surgery, Division of Veterinary Research, Obihiro University of Agriculture and Veterinary Medicine, Hokkaido 080-8555, Japan; anco@vet.ne.jp; 5 Department of Internal Medicine, Faculty of Veterinary Medicine, Uludag University, Bursa 16120, Turkey; zyilmaz@uludag.edu.tr; 6 Department of Pediatrics and Adolescent Medicine, Juntendo University Graduate School of Medicine, Tokyo 113-8421, Japan; myifuku@juntendo.ac.jp (M.I.); takeshii@juntendo.ac.jp (T.I.); kentaka@juntendo.ac.jp (K.T.)

**Keywords:** cardiomyopathy, dogs, doxorubicin, echocardiography, heart failure, intraventricular pressure gradient, pressure-volume

## Abstract

**Simple Summary:**

Cardiotoxicity is a serious side effect of doxorubicin in cancer patients due to the risk of development of heart failure. Early detection of doxorubicin-induced cardiomyopa-thy (DXR-ICM) has become a major objective to reduce heart failure in cancer patients. Echocar-diography is the gold standard method to diagnose cardiac diseases when cardiac dysfunction is prominent; however, it still cannot predict or early diagnose heart failure before functional de-cline. The intraventricular blood flow is characterized by intraventricular pressure gradients (IVPG) that created due to the suction of blood by the ventricles. Currently, advanced imaging techniques allow non-invasive assessment of IVPG from color M-mode echocardiography (CMME) after image processing for the clinical setting. Studies revealed that IVPG indices are promising for the early diagnosis of cardiac dysfunction. In this study, we aimed to investigate the usefulness of IVPG to detect cardiac function changes after DXR administration in dogs.

**Abstract:**

Early detection of doxorubicin (DXR)-induced cardiomyopathy (DXR-ICM) is crucial to improve cancer patient outcomes and survival. In recent years, the intraventricular pressure gradient (IVPG) has been a breakthrough as a sensitive index to assess cardiac function. This study aimed to evaluate the usefulness of IVPG for the early detection of chemotherapy-related cardiac dysfunction. For this purpose, six dogs underwent conventional, speckle tracking, and color M-mode echocardiography concomitantly with pressure-and-volume analysis by conductance catheter. The cardiac function measurements were assessed before DXR administration (baseline, Pre), at the end of treatment protocol (Post), and at 1.5 years follow-up (Post2). The result showed a significant reduction in the left ventricular end-systolic pressure-volume (Emax: 4.4 ± 0.7, 6.1 ± 1.6 vs. 8.4 ± 0.8 mmHg/mL), total-IVPG (0.59 ± 0.12, 0.62 ± 0.15 vs. 0.86 ± 0.12 mmHg), and mid-IVPG (0.28 ± 0.12, 0.31 ± 0.11 vs. 0.48 ± 0.08 mmHg), respectively in Post2 and Post compared with the baseline (*p* < 0.05). Mid-to-apical IVPG was also reduced in Post2 compared with the baseline (0.29 ± 0.13 vs. 0.51 ± 0.11). Meanwhile, the fraction shortening, ejection fraction, and longitudinal strain revealed no change between groups. Total and mid-IVPG were significantly correlated with Emax (R = 0.49; *p* < 0.05, both) but only mid-IVPG was a predictor for Emax (R^2^ = 0.238, *p* = 0.040). In conclusion, this study revealed that impairment of contractility was the initial changes observed with DXR-ICM in dogs and only IVPG could noninvasively detect subclinical alterations in cardiac function. Color M-mode echocardiography-derived IVPG could be a potential marker for the early detection of doxorubicin cardiomyopathy.

## 1. Introduction

The extended life expectancy of cancer patients who have received treatment is clearly a positive development; however, the drawbacks of development of heart failure (HF) due to the cardiotoxic activity of the chemotherapy, for instance, doxorubicin (DXR)-induced cardiomyopathy (DXR-ICM), has become a serious problem for patients who have survived the neoplastic disease. Despite the effectiveness of DXR in chemotherapy, the increased likelihood of cardiotoxic activity of the drug, which determines the patient’s survival, becomes a management challenge for oncologists. Studies have revealed that DXR affects cardiac function in a cumulative and dose-dependent manner in humans and dogs [1,2]. After 10 years of follow-up, cumulative doses of anthracycline (more than 500 mg/m^2^) resulted in left ventricular dysfunction in 63% of patients compared with 18% prevalence in patients who received less than 500 mg/m^2^ [3]. Additionally, dogs receiving DXR at a cumulative dose rate of 144.8 mg/m^2^ showed clinical cardiotoxicity [2]. The mortality rate in patients with DXR-ICM is significantly higher compared to other cardiomyopathy patients. Indeed, only 40% of patients who underwent DXR therapy survived up to two years following the onset of HF [4]. Regardless of the usefulness of DXR in patients with malignant tumors associated with poor survival rates, its adverse effects limit its use as aggressive therapeutic options. Therefore, the early and accurate detection and/or prediction of the onset of the expected HF become crucial for improving the clinical implication purposes of DXR in cancer patients.

The ejection fraction (EF) in human medicine and fractional shortening (FS) in veterinary practice are widely used indices in echocardiographic examination to evaluate cardiac function [5]. However, these indices are not sensitive enough to detect the early deterioration in contractility resulting from DXR-ICM. In this regard, a reduction in EF in cancer patients, which is more likely to appear in the advanced stage, indicates the development of cardiac dysfunction that would interrupt the continuity of the chemotherapy protocol and subsequently impacting the survival outcome [6]. Therefore, there is an urgent need to discover a novel and sensitive index to enable earlier detection of chemotherapy-related cardiac dysfunction. 

Although most attention has been paid to systolic function in the diagnosis of DXR-ICM, several reports have suggested that diastolic dysfunction precedes systolic failure in both human and canine patients [7,8,9]. Recently, the measurement of diastolic function has therefore begun to attract more attention in hopes for the early detection of cardiac events. The intraventricular pressure difference (IVPD) during early diastole is an index that focuses on intraventricular blood flow, and is considered a sensitive indicator of diastolic function. IVPD is obtained from color M-mode echocardiographic (CMME) images using Euler’s equation [10]. The intraventricular pressure gradient (IVPG) can be calculated by dividing the IVPD by the length of the left ventricle (LV); consequently, unlike the IVPD, IVPG is not affected by the size of the heart [11,12,13]. Generally, the length of the LV, from the mitral valve to the apex, is divided into basal, mid, and apical parts, and each has its corresponding IVPG index. The mid to apical-IVPG, which is the pressure difference between mid and apical parts, represents a suction force that is reported to reflect the active relaxation of the LV during diastole. Besides, Tau, which is the most established index to describe left ventricular diastolic function that obtained invasively by catheterization, showed a negative correlation with total-, basal-, and mid-IVPD values in an experimental canine model under different loading conditions [14]. 

Recently, the evaluation of diastolic and systolic IVPG has shown promising results in the evaluation of heart functions and showed agreeable feasibility and reproducibility in humans [15]. The clinical applicability of the IVPG in both healthy and in patients with diastolic HF indicated the inability of the heart to produce an effective ventricular suction power under stress conditions [16,17]. These studies have used the same approach to non-invasively evaluate the IVPG through CMME. Currently, conventional imaging techniques and biomarkers cannot accurately detect DXR-cardiotoxicity before functional decline [18,19,20,21]. Therefore, this approach is expected to provide a useful new index to overcome the shortcoming of the conventional echocardiographic indices, particularly in early diastole interpretation, and the incorporation of this technology in clinical oncology may reflect its usefulness for early diagnosis of HF which is necessary to allow quick medical interference. Therefore, in the present study, we aimed to measure sequential changes in the flow indices and evaluate the utility of the IVPG for the early detection of subclinical cardiac dysfunction in dogs after DXR administration. 

## 2. Materials and Methods

### 2.1. Animals and Study Protocol 

Six adult female beagle dogs (Kitayama Labes, Nagano, Japan) weighing between 8–10 kg (9.3 ± 0.8 kg, mean ± SD), 2 to 3 years old, were used in this study. All dogs were physically fit based on the medical examination and routine hemato-biochemical profile. Special attention was paid to the cardiac function, and the dogs were free from any cardiac abnormalities upon auscultation of the heart, electrocardiography, and echocardiography. All dogs were administered six consecutive doses of DXR (30 mg/m^2^) via the cephalic vein at three weeks intervals [2], and the cumulative dose was restricted to 180 mg/m^2^. Echocardiography and catheter measurements were performed at three-time intervals: before DXR administration (Pre), after completing the treatment at 4.5 months (Post), and 1.5 years post-treatment (Post2). This study was conducted under strict anesthetic doses and stable hemodynamic conditions for all dogs across the study to exclude the effect of anesthesia on the obtained measurements.

### 2.2. Anesthesia and Preparatory Procedure 

The dogs were pre-medicated with atropine (0.05 mg/kg, SC, Tanabe Seiyaku Co. Ltd., Hackensack, NJ, USA), meloxicam (0.2 mg kg^1^; Metacam 0.5% injectable; Boehringer Ingelheim Vetmedica Japan, Japan), and ampicillin-sodium (20 mg/kg, Ampicillin Na, Fujita-Pharm, Tokyo, Japan) in addition to buprenorphine (0.01 mg/kg, Lepetan Injection, Otsuka Pharmaceutical Co. Ltd., Tokyo, Japan) to provide an analgesic effect. Anesthesia was induced by propofol (4 mg/kg IV, Propofol 1% Injection, Pfizer Japan Inc) and then maintained by isoflurane (end-tidal concentration of 1.5 ± 0.1 %, Fujifilm Wako Pure Chemical Corporation, Japan) after tracheal intubation. All dogs were administered lactated Ringer’s solution (5 mL/kg, CIV, Lactec Injection, Otsuka Pharmaceutical Co. Ltd.). The respiratory rate was controlled by an anesthesia machine (positive pressure ventilation). Expiratory CO_2_ was controlled at 35–45 mmHg. 

### 2.3. Catheter Examination

All dogs were placed in left lateral recumbency, and the right common carotid artery was exposed from the adjacent tissue, and a 6-Fr sheath was introduced into the lumen. A conductance catheter (Ventri-Cath-507, Millar Instruments, Houston, TX, USA) was inserted into the LV through the sheath being guided by fluoroscopy. The analysis of real-time pressure-and-volume loop data was conducted using the Micro-Tip Pressure-Volume Ultra Foundation System (MPVS-Ultra-S, AD Instruments, Dunedin, New Zealand), PowerLab hardware (ML880 PowerLab 16/30, AD Instruments), and LabChart Pro software (LabChart v7, AD Instruments). LV end-diastolic pressure and Tau were calculated from 10 consecutive pressure-and-volume loops. Tau was measured according to the method of Weiss et al. [22]. The values of Emax and stiffness constant β were determined using the previously described method [23]. Emax was measured by linear regression of the end-systolic pressure-volume relation (EDPVR) during transient caval occlusions from six successive pressure-volume loops [24]. Stiffness constant β was obtained using the exponential equation of the EDPVR during the same procedure as Emax [25] as follows:

EDP = Ae ^β·EDV^

where EDP is the end-diastolic pressure, EDV is end-diastolic volume, and A is the curve-fitting constant. 

### 2.4. Conventional and Speckle Tracking Echocardiography

The echocardiographic assessment was performed using a Prosound F75 with a sector probe of 5 MHz (Hitachi-Aloka Medial, Tokyo, Japan). All dogs were placed in lateral recumbency, and the echocardiography was performed after confirming stable hemodynamics and equable conditions for 10 min after catheterization. Three heartbeats were recorded at the end of the expiratory phase. The LV inner diameter at end-diastole (LVIDd), EF, and FS were calculated in the short-axis view at the papillary muscle level through M-mode [26]. The LV inflow wave pattern was measured in the left apical long-axis four-chamber view, and the early (E) and late (A) velocities, E/A ratio, and deceleration time (DecT) were recorded. The tissue Doppler imaging (TDI) indices (é, á, é/á, E/é ratio) were also acquired after the cursor overlapping the base of the mitral valve. 

For two-dimensional speckle tracking echocardiography (2DSTE), motion images from the left parasternal long-axis view were obtained with the frame rates of 70–110 frames/s and saved for further off-line analysis using the special software (DAS-RS1 software 1.1v, Hitachi Aloka Medical, Tokyo, Japan) to measure the global longitudinal strain (GLS) and the early diastolic strain rate (EDSR). Both basal- and apical-level images of the right parasternal short-axis view were used to obtain the LV twist. 

### 2.5. Color M-Mode Echocardiography for Assessment of IVPG 

**Figure 1:**
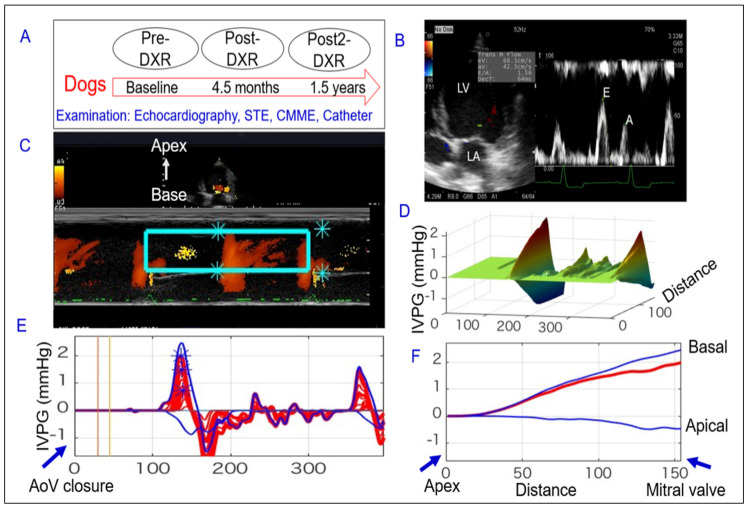
Schematic representation of the used procedures to evaluate cardiac function during doxorubicin-induced cardiomyopathy in dogs. Catheterization and different echocardiographic approaches (conventional, two-dimensional speckle tracking (2DSTE), color M-mode echocardiography (CMME)) were carried out at three time-points; Pre, Post, and Post2-doxorubicin (DXR) administration (**A**). After optimizing the mitral inflow from the left apical view (**B**), CMME was switched on to trace the inflow tract from the left atrium (LA) to the apex of the left ventricle (LV). CMME images (**C**) were used for IVPG assessment after determination of the area of interest (mitral inflow, rectangular green box) using MATLAB software, by which the 3D temporal and spatial profile of IVPG (**D**), and IVPG time distribution (**E**,**F**) were calculated using Euler’s equation.

The intraventricular pressure gradient (IVPG) was calculated from the image obtained from the CMME of the left parasternal longitudinal apical four-chamber view. The machine was set to sweep speed of 300 mm/s and color baseline-shift of 64 to increase the Nyquist limit for proper tracing of the CMME. The IVPG was calculated using in-house code written in MATLAB (The MathWorks, Natick, MA, USA), as showed in Figure 1. Previously, IVPD derived from CMME was validated against the temporal IVPD obtained by the catheterized method using a micromanometer in animal experiments [16]. The IVPG values were derived from the IVPD according to the following formula: IVPG (mmHg/cm) = IVPD/LV length. 

The LV was divided into three equal parts. The basal part was defined as the region comprising one-third from the mitral valve to the apex. The middle part was defined as the mid part, and the remaining part was defined as the apical part. Besides, a two-thirds segment of LV length on the apex side was adopted as mid to apical-IVPG [27]. IVPG analysis was conducted for three heartbeats at the end of the expiratory phase, and the mean value was used for the statistical analysis.

### 2.6. Statistical Analysis

All statistical analyses were performed using R software (version 3.6.0, The R Foundation). Data were reported as mean ± SD. For all statistics, *p* values < 0.05 were considered statistically significant. After an assessment of the differences in the Pre, Post, and Post2 results using one-way repeated-measures ANOVA, Tukey’s honestly significant difference post hoc test was used to compare variables between the Pre, Post, and Post2 results. Pearson’s correlation coefficients were used to assess the relationship between Emax and conventional, 2DSTE, and the IVPG variables. Multivariate linear regression analysis was performed to determine the independent variables that correlated with Emax. The stepwise method was used for the selection of variables.

## 3. Results

### 3.1. Pressure-Volume Analysis by Catheterization

Table 1 shows the pressure-volume analysis indices for the Pre-, Post- and Post2-DXR administration. Stiffness constant β, Tau, and LV end-diastolic pressure values revealed no significant difference throughout the experimental intervals. Meanwhile, Emax was significantly lower in the Post and Post2-stages compared with Pre-stage (6.1 ± 1.6 and 4.4 ± 0.7 vs. 8.4 ± 0.8 mmHg/mL; *p* < 0.01, 0.001), respectively. The Emax was reduced with time course after medication and also found to be significantly lower in Post2-stage than Post (4.4 ± 0.7 vs. 6.1 ± 1.6 mmHg/mL; *p* < 0.05).

### 3.2. Conventional and Two-Dimensional Speckle Tracking Echocardiographic Indices

The conventional and two-dimensional speckle tracking echocardiographic indices are summarized in Table 2. There were no significant differences in LVIDd, EF, FS, diastolic LV velocity indices (Peak E, Peak A, E/A, and E-wave deceleration time), as well as the TDI velocity indices (ś, é, á, E/é) across the defined experimental intervals (*p* > 0.05). There was no significant change in 2DSTE indices either.

### 3.3. Intraventricular Pressure Gradients (IVPG) Analysis

**Figure 2:**
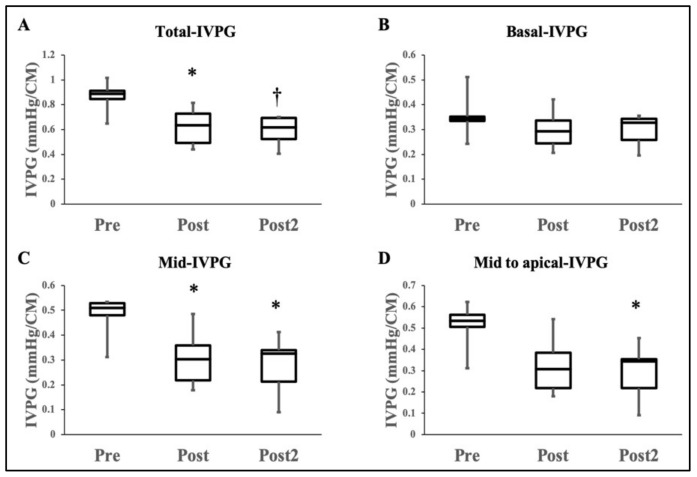
Box plots of the distribution of the IVPG indices. Data presented as mean ± SD. Pre is before administration of DXR administration. Post is after the end of DXR administration. Post2 is 1.5 years after complete medication. The total-IVPG was decreased significantly in the Post and Post2-measurements than Pre-measurement (**A**). Basal-IVPG was not significantly changed (**B**). The mid-IVPG was decreased significantly in the Post and Post2-measurements than Pre-measurement (**C**). The mid to apical-IVPG was impaired significantly in the Post2-measurement than Pre-measurements (**D**). * *p* < 0.05 and † *p* < 0.01 refer to comparisons between the Pre-measurement and Post, Post2-measurements.

Figure 2 shows the results of changes in the IVPG indices before and after DXR administration. The total-IVPG “from left atrium to the LV apex” was significantly decreased in the Post and Post2-stages compared with the Pre-stage (0.62 ± 0.15 and 0.59 ± 0.12 vs. 0.86 ± 0.12 mmHg; *p* < 0.05, 0.01; respectively). Similarly, mid-IVPG was significantly decreased in the Post and Post2-stages compared with Pre-stage (0.31 ± 0.11 and 0.28 ± 0.12 vs. 0.48 ± 0.08 mmHg; *p* < 0.05). Besides, there was a significant reduction in mid to apical-IVPG in the Post2-stage compared with Pre-stage (0.29 ± 0.13 vs. 0.51 ± 0.11; *p* < 0.05). In contrast, basal- and apical-IVPGs were relatively constant throughout the experimental time intervals.

### 3.4. Relationship between Emax and Conventional, Two-Dimensional Speckle Tracking Echocardiographic, and the IVPG Indices

**Figure 3:**
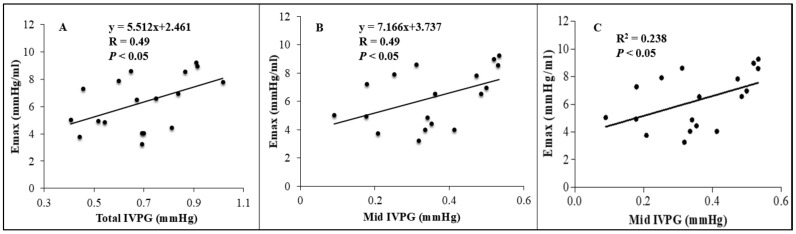
(**A**,**B**) Pearson’s correleation between changes in Emax: left ventricular end-systolic pressure-volume, and total- and mid-IVPG: intraventricular pressure gradient showed significant positive correlation (r = 0.49, *p* < 0.05, both (**A**,**B**)). (**C**) linear regression analysis of Emax and mid-IVPG revealed that mid-IVPG was a predictor of Emax (R^2^ = 0.0.238, adjusted R^2^ = 0.190, *p* < 0.05).

There was no correlation between Emax and conventional, and 2DSTE indices. On the other hand, as observed in Figure 3, total-IVPG and mid-IVPG showed a significant positive correlation with Emax (r = 0.49, *p <* 0.05, both). Meanwhile, no significant correlation between Emax and basal-, apical-, and mid to apical-IVPGs were observed. The multivariate analysis determined that only mid-IVPG was a predictor of Emax (adjusted *R*^2^ = 0.190, *p* < 0.05), as shown in Figure 3C.

## 4. Discussion

Side effects of chemotherapy are an endless challenge that has attracted comparative oncologists to use dogs as models to evaluate the pros and cons of therapeutic strategies [28,29]. Cardiotoxicity is a common drawback of the DXR and subsequently leads to cardiomyopathy in cumulative dosing in dogs [2]. Several studies have shown diastolic dysfunction to be the earliest manifestation of DXR-ICM [7,8,9,30]. However, it is not easy to prove that the impairment of diastolic function is the earliest stage of HF for several reasons. For instance, in studies using laboratory animals, the experimental interventions are often so drastic that the DXR-related cardiac changes are evident in both systole and diastole [31,32]. Furthermore, for clinical application, it is still hard to accurately interpret diastolic cardiac function as conventional echocardiography has its limitation and invasive catheterization is not clinically applicable [33,34]. To the best of our knowledge, no studies have been conducted to observe how the impairment of cardiac function occurs just after the onset of DXR-ICM. Thus, in the present work, we conducted for the first time a long-term study to monitor the cardiac function changes through the widely used conventional echocardiographic indices (EF, FS, and GLS) concomitantly with novel and non-invasive indices (IVPG), and compare the IVPG results with the gold standard indices obtained by invasive catheterization following the experimental administration of DXR.

In this study, EF and FS did not show a statistically significant decline throughout the experiment in comparison to the reference values (39.63 ± 6.26 and 70.9 ± 6.0), respectively [35], and could not exactly detect the deterioration in cardiac contractility as previously reported [19]. However, other studies in dogs reported a significant reduction in FS with a similar dose of DXR [31,36,37]. These studies were conducted using dogs that were more mature and/or suffering from neoplastic disease, whereas the present study involved young healthy dogs. Thereby, FS may have declined earlier in these animals in the other studies. Furthermore, echocardiographic methods such as trans-mitral flow velocity pattern, tissue Doppler imaging (TDI), and 2DSTE were previously valued as reliable indices for the evaluation of cardiac dysfunction. However, in the present study, no changes could be detected using these conventional methods. Myocardial injury can largely depend on age and remaining capacity to resist DXR toxicity [38]. Therefore, the degree of myocardial injury in subclinical cardiomyopathy, as in the present study, may have been relatively mild that it cannot be detected by EF, FS, and 2DSTE.

The current study revealed that the deterioration of systolic function precedes the impairment of diastolic function in DXR-ICM. Only Emax obtained by invasive catheter was able to detect impairment in left ventricular contractility. On the other hand, Tau “LV diastolic time constant”, the most widely used index for ventricular relaxation, showed no obvious change during the study period.

Several reports have suggested that diastolic dysfunction precedes systolic failure. This is contrary to recent studies suggesting that diastolic indices may be the earliest predictors of DXR-ICM [9,30]. Here, we have to take into consideration that EF and FS represented contractility in these studies. However, these parameters showed no change after DXR administration [19] and some researchers consider these indices are not sensitive enough to detect early deterioration in contractility in DXR-ICM [6,21,39]. Since EF can only detect changes once myocardial damage had already begun [31]; therefore, it must be carefully interpreted as a contractility index in terms of its sensitivity.

Data of the currently used indices of contractility are controversial. GLS, which focuses on LV contractility in the long axis direction, was reported as a sensitive indicator even when EF was preserved [40], but its data relating to DXR-associated cardiotoxicity are not entirely consistent across the studies. For instance, some studies showed its feasibility after a long time of follow-up in human patients (median 23 years) [39,40,41]. Besides, a previous study that relied on reduced GLS and EF (as indicators of myocardial function) failed to early diagnose subclinical cardiomyopathy and to establish an efficient cardioprotective protocol in anthracycline-treated patients because of delayed changes in these parameters, in addition to their ability to diagnose cardiomyopathy only in worsen cases due to a combination of anthracycline and trastuzumab [39,42]. Also, GLS has shortcomings including, but not limited to, high inter-observer variability, improper imaging, and temporal resolution [43,44]. In other studies, which used EF and FS as predictors of deterioration in contractility, there was a possibility that the potential impairment of contractility could not be detected because of the poor sensitivity of conventional methods compared with invasive measurement, even though a decrease in contractility may have already occurred. For these reasons, undoubtedly, a more sensitive diagnostic index is urgently needed for saving non-responder patients [45]. Thus, our findings stress the importance of detecting a preceding deterioration in systolic function in DXR-ICM by measuring the IVPG.

During the early diastolic period, the LV works as an effective pump, actively drawing blood from the left atrium into the LV apex, without any elevation of left atrial pressure. This suction force created by the pressure gradient from the left atrium to the LV apex is a major determinant of adequate filling in the early diastole during normal cardiac function [16,45,46]. The IVPG has been recognized as an index of diastolic function in early diastole [14]; however, in the present study, IVPG was decreased even though the diastolic function was preserved as indicated by the non-significant change in Tau and left ventricular end-diastolic pressure. At the beginning of relaxation, the energy stored in the LV wall during systole is released [47]. This step is referred to as “elastic recoil”. At the same time, ATP-consuming actin-myosin decoupling, known as “active relaxation,” also occurs. While basal-IVPG reflects left atrial pressure, mid-and mid to apical-IVPG are affected by both elastic recoil and active relaxation [45]. In the present study, the impairment of contractility may have been the reason for the mid-IVPG decline that may be developed due to a decrease in elastic recoil. This is consistent with a study that showed a linear correlation between the IVPG and Emax in experimental dog models [48]. Although it was an unprecedented finding that the mid-IVPG could reflect the decline in elastic recoil without documenting any change in Tau, the IVPG may indicate early impairment of contractility after chemotherapy even if it is too mild or subclinical to be detected via conventional measurements. This result suggests that the mid-IVPG can be a useful index during diastole that reflects contractility with great sensitivity and reflecting the close relationship between contraction and relaxation.

Attention has been paid to the evaluation of diastolic function based on the prior decline of diastolic function. The quantitative evaluation of therapeutic effects and the progression of HF is complicated. The 2016 American Society of Echocardiography and European Association of Cardiovascular Imaging guidelines for diastolic function assessment have recommended an evaluation that combines multiple indices instead of a single index because of their dependency on age and preload [49]. Although EF is used first to differentiate between HF with preserved and reduced EF, this quantitative assessment is not valued in evaluating the severity of HF because it is thought that patients with reduced EF also have impaired diastolic function. Furthermore, the use of GLS remains a supplementary index for the measurement of myocardial function. The present study revealed the preceding impairment of systolic function and the high sensitivity of the IVPG for evaluating contractility. The diastolic function of IVPG is very meaningful, but if more research studies investigate the utility of IVPG assessment in heart contractility interpretation, an improved prognosis for patients with DXR-ICM might be possible by earlier therapeutic interventions with cardioprotective agents.

### 4.1. Clinical Implications

Although there are some limitations in IVPG assessment, including the inter-system variability, IVPG is expected to be superior to GLS because it provides a higher frame rate to illustrate the heart with good feasibility and reproducibility [15]. In this study, we showed that IVPG assessment has a committed value in detecting the earliest alteration of cardiac function due to DXR administration among EF, FS, and GLS indices. This study also described that the deterioration of systolic function precedes the impairment of diastolic function measured by pressure-volume analysis using a conductance catheter. Despite IVPG assessment is still being in infancy, cardiologists are continuously seeking fruitful values for its clinical implication, and we thought that the utility of IVPG for the early detection of DXR-ICM could be achieved.

### 4.2. Limitations

The time lag between Post and Post2 was relatively long because of the unrepeatability and unacceptability of invasive catheterization. The study period was relatively small to observe overt cardiac dysfunction, but DXR-ICM is variable and some patients develop acute toxicity, while others may have no symptoms for 10 years [28]. This point may be covered by DXR overdosing, but it would not be clinically approved. The number of dogs was also relatively small, but the normality of the produced data was acceptable, and our team showed good repeatability of the used techniques [11]. Besides, we did not study the changes in IVPG under anesthesia since a stable hemodynamic condition was followed in the used protocol. However, the similarities in human cardiac physiology with canine IVPG, supported by other studies, suggest that our principle is valid for investigating the effects of doxorubicin in IVPG [50]. The relationship between the IVPG and cardiac biomarkers also did not address and necessitate further studies.

## 5. Conclusions

Invasive measurements over multiple time intervals suggested that impairment in systolic function precedes decreasing diastolic function following the administration of DXR. A progressive decline in elastic recoil of the LV wall during the systolic period may lead to a decrease in the IVPG following a deterioration in the suction force. The IVPG may be the key to promote the early detection of DXR-ICM from the novel perspective that the IVPG is not only an index of diastolic function but also a potential sensitive marker of contractility in subclinical cardiac toxicity.

## Figures and Tables

**Table 1:** Pressure-volume analysis measurement by invasive catheterization in dog model of DXR-ICM.

Indices	Unit	Pre	Post	Post2	*p*-Value
Stiffness constant β	mmHg/mL	0.09 ± 0.03	0.13 ± 0.04	0.15 ± 0.05	0.07
Tau	ms	32 ± 9	36 ± 8	37 ± 6	0.57
LV end-diastolic pressure	mmHg/mL	11 ± 7	13 ± 5	10 ± 3	0.67
Emax	mmHg/mL	8.4 ± 0.8	6.1 ± 1.6 *	4.4 ± 0.7 ^†‡^	<0.001

Mean ± SD of the cardiac function parameters obtained by invasive catheterization. * *p* < 0.01 vs. Pre. ^†^ *p* < 0.001 vs. Pre. ^‡^ *p* < 0.05 vs. Post. Emax: left ventricular end-systolic pressure-volume, Tau: Left ventricular diastolic time constant.

**Table 2:** Conventional and two-dimensional speckle tracking echocardiographic indices throughout the experimental time intervals during DXR-ICM in dogs.

Indices	Unit	Pre	Post	Post2	*p*-Value
Conventional indices					
HR	pbm	112 ± 12	111 ± 10	105 ± 14	0.52
LVIDd	mm	30 ± 1	32 ± 1	33 ± 4	0.28
EF	%	78 ± 5	72 ± 9	70 ± 11	0.13
FS	%	40 ± 5	36 ± 7	34 ± 8	0.15
E velocity	cm/s	76 ± 10	67 ± 9	69 ± 10	0.29
A velocity	cm/s	48 ± 16	43 ± 15	50 ± 12	0.61
E/A		1.8 ± 0.8	1.7 ± 0.6	1.4 ± 0.3	0.31
E DecT	ms	86 ± 9	86 ± 14	91 ± 30	0.88
ś	cm/s	8.6 ± 1.3	8.0 ± 1.6	7.5 ± 0.5	0.18
é	cm/s	9.5 ± 1.3	8.0 ± 1.7	8.2 ± 1.7	0.23
á	cm/s	8.0 ± 2.4	7.2 ± 2.2	7.1 ± 1.1	0.66
E/é		8.0 ± 1.0	8.7 ± 1.2	8.9 ± 1.4	0.26
Two- dimensional speckle tracking echocardiography indices					
GLS	%	17 ± 3	15 ± 2	16 ± 3	0.57
EDSR	1/s	2.1 ± 0.6	1.6 ± 0.4	2.0 ± 0.4	0.22
LV twist	°	8.1 ± 3.5	5.8 ± 2.8	5.4 ± 1. 2	0.07

The echocardiographic parameters were obtained from conventional and speckle tracking imaging techniques in dogs. Data expressed as mean ± SD. HR, heart rate; LVIDd, LV inner diameter at diastole; EF, ejection fraction; FS, fraction shortening; E velocity, early diastolic velocity mitralis; A, late diastolic velocity mitralis; E/A, early to late mitral inflow velocity ratio; E DecT, deceleration time; ś,systolic velocity of the LV wall; é, early diastolic velocity of the LV wall; á, late diastolic velocity of the LV wall; E/é, early diastolic velocity mitralis to the early diastolic velocity of the LV wall ratio; GLS, Global longitudinal strain; EDSR, Early diastolic strain rate.

## Data Availability

The data presented in this study are available on request.
